# Editorial

**Published:** 2016-07-09

**Authors:** Rauf M Aghayev

**Affiliations:** Pofessor, Department of Surgical Diseases-II, Azerbaijan Medical University, Baku, Azerbaijan, Euroasian Journal of Hepato-Gastroenterology

Dear Colleagues,

This year marks the 20th anniversary of establishment of the Eurasian Association of Gastroenterology (EAG). Decision about establishing this society was carried in 1996 in Antalya (Turkey) during the days of Turkish National Congress of Gastroenterology, when some famous Turkish scientists and participants from Turkish-origin foreign countries discussed about need in some society for closer scientific contacts between gastroenterologists and surgeons of their countries. The organization of such a scientific association with headquarters in Ankara has been approved by all participants of a meeting and the charter of new society has been prepared and approved soon. Turkey, Azerbaijan, Kazakhstan, Uzbekistan, Kyrgyzstan, Turkmenistan, and Northern Cyprus became full members of the created society. According to the charter, main aims and goals of new society were establishment of scientific cooperation, the constant exchange of ideas, and experience among scientistsgastroenterologists and surgeons from these countries, familiar with one another’s achievements, such as help for improvement of professional levels of doctors-specialists. For achievement, these goals were decided carrying the Congresses of EAG no later than once a year in one of the member countries of Association. The first International Euroasian Congress has been carried out in 1997 in the capital of Azerbaijan-- Baku city.

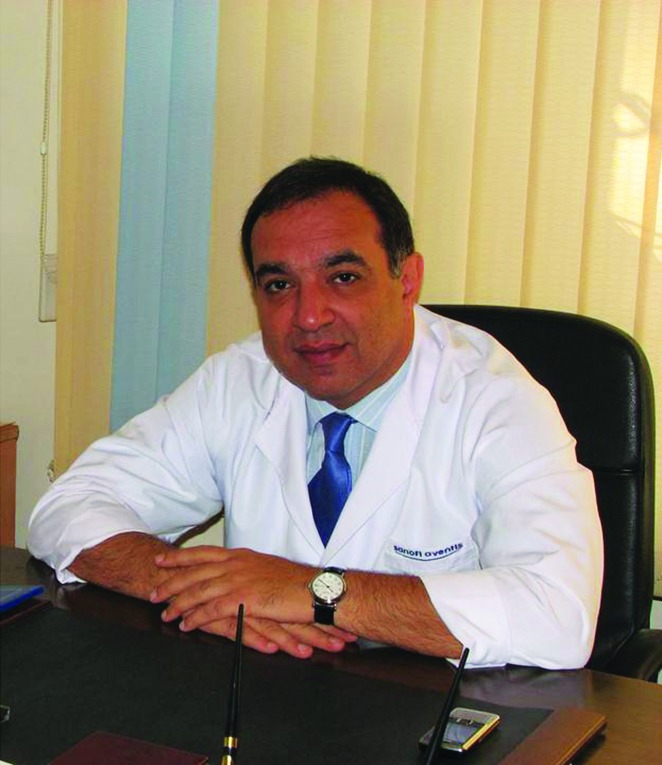



The Congress that was carried out with assistance of the government of Azerbaijan had 35 scientists from five foreign countries and about 500 Azerbaijani doctors. Upon completion of Congress, representatives from member countries of new Association have noted the success of the held forum and have emphasized the importance of such meetings for specialists of their countries.

In the next years, the Euroasian Congresses of Gastroenterologists and Surgery was carried out in Kazakhstan (1998), Turkey (1999), Uzbekistan (2000), Kyrgyzstan (2001), and again in Azerbaijan (2003).

In 2004, Professor of Ankara University, Hasan Ozkan was elected as the President of the Association and till date he successfully directs this scientific organization. It should be noted that in the next Euroasian Congress of Gastroenterologists in Ankara held in 2004, the meeting was held between the new management of Association with members of the international scientific committee of society where a number of important questions were discussed.

So, during the meeting participants had expressed opinion about the necessesity of some changes in a format of the held scientific forums. It has been noted that scientists from other non-Turkish-origin countries show a great interest to scientific Congresses of EAG and they repeatedly address to presidium of society about inclusion of their countries in Association like full members of society. Considering it, full members of Association in the next years will be elected in Ukraine, Georgia, Ýran, Russia, Bangladesh, Bulgaria, Pakistan, Bosnia and Herzegovine, Egypt, Albania, Moldova, and other countries. For this reason, the next Congresses of EAG have been carried successfully out in the countries which became new members of the organization—Georgia (2005) and Ýran (2007).

The Azerbaijani surgeons and gastroenterologists are one of the most active participants of all events held by EAG from the moment of its establishment. The big and irreplaceable role in formation of EAG and its work belongs to the famous Azerbaijani scientist- academician Beyukkishi Agayev. Under his management in the capital of Azerbaijan, the Congresses of EAG have been carried out six times (1997, 2003, 2006, 2008, 2011, and 2013).

In Azerbaijan as well as in other countries, interest of doctors to these significant scientific meetings has grown from year to year that is a graphic evidence of the acquired authority of EAG on the scientific world. So, in the first Congress held in Baku, there were 35 foreign scientists from five countries; whereas in the Congress held in 2013, the foreign delegation was presented by 162 doctors representing the leading medical clinics and universities of 25 countries. Their articles and lectures about modern problems of surgery and gastroenterology, information about new views on diagnostics, and treatment of a number of diseases had a huge value for all participants of the Congress and of course for the young Azerbaijani doctors and residents who were taking the first steps in science.

In these Congresses, together with the well-known Azerbaijani scientists (which held in Baku in different years) famous scientists from different countries also took part: H. Özkan, A. Dökmeci, S. Ünal, N. Örmeci, Ý. Balik, Y. Bayraktar, S. Yýlmaz (Turkey) A. Nakao, F. Akbar, H. Miroyama (Japan), R. Gish, S. Gupta (USA), M. Farthing (UK), F. Ezzat, A. Sami (Germany), N. Lygidakis (Greece), M. Abdel Vahab (Egypt), E. Galperin, V. Sandrikov (Russia), A. Hacibayev (Uzbekistan), E. Gerich (Ukraine), M. Al Mahtab (Bangladesh), G. Parodi (Italy), J.Heyat (Iran), and others.

These Congresses were widely highlighted on local television, on pages of newspapers, scientific journals, and the web sites. A number of participants of the Congress have had official meetings with heads of the Ministry of Health of the Republic and the Azerbaijan Medical University. During these meetings, the Azerbaijani heads emphasized value and role of such scientific forums for scientists and doctors of Azerbaijan, about the need of close contacts among experts for increasing their professional level.

Azerbaijani Association of Surgeons and Gastroenterologists (AASG) established in 1997 played a big role in formation and development of surgery and gastroenterology in Azerbaijan. AASG unites more than 1,000 surgeons and gastroenterologists who work in the leading clinics of Baku, scientific institutes, regional hospitals, and polyclinics. The association holds permanent meetings, conferences, and symposiums with participation of foreign experts, where topical issues on diagnostics and treatment of various diseases are discussed. Much attention is paid on questions of prevention and modern methods of treatment of viral hepatitis, surgical and endoscopic treatment of the biliary strictures, parasitic cysts of a liver, a hepatocellular carcinoma, and so on. Because of activity of the AASG, doctors have an opportunity for being informed constantly about the achievements of medicine, new methods of diagnostics, and alternative ways of treatment of various diseases.

With an initiative and support of AASG, a number of doctors from Azerbaijan take advanced training courses in the leading clinics of Baku and foreign countries. In 2005, the Association started the journal “Cerrahiyye” (Surgery) whose editorial board includes scientists from 16 countries. The journal publishes materials not only in Azerbaijani, but also in English and Russian languages, so scientists from various countries of Europe, Asia and America have published their articles in journal of AASG.

Azerbaijani surgeons and gastroenterologists is playing an important role in cooperation between AASG with EAG. This cooperation allowed a great number of young doctors from Azerbaijan to have scientific trainings and increasing the professional level in the leading clinics of Turkey. With assistance of our Turkish colleagues, a lot of qualified specialists who in recent years have been trained in Ankara, Istanbul, and Izmir are now actively working in the leading clinics and scientific divisions of Azerbaijan institues.

In Antalya (Turkey) from April 27th to May 1st of 2016 has passed the XV Congress of Euroasian Gastroenterologists and Surgeons under the chairmanship of the president of EAG, Professor H. Özkan. In the Congress that was organized at a very high level, guests from various countries have highly appreciated activity of society and have emphasized its role in the development of science and preparation of high-quality specialists. In Antalya, they disscussed some prospects of further works and taken a decision about carrying out the next EAG Congress in 2017 in Sarajevo. I am sure that this Congress in the territory of ancient Bosnia will open new prospects in activity of EAG and will serve expansion of structure of Association at the expense of the new countries from the European region.

I want you all to note that Euroasian Journal of Hepato-Gastoenterology is playing a very important role in collaboration and connection among scientists from the Euroasian countries and increasing of the EAG’s authority in the scientific world since 2011. In a short period, this journal has become very popular among readers from many countries, including Azerbaijan. Here of course is a big merit of Editor-in-Chief of the journal, Prof Hasan Özkan, co-Editor-in-Chief, Prof Fazle Akbar, and Executive Editor, Prof. Mamun-Al-Mahtab.

I want to wish all my colleagues a very good health and success in their professional activity and to wish EAG and Editorial board of EJOHG prosperity and further strengthening of the international scientific authority.

**Rauf M Aghayev**

Pofessor, Department of Surgical Diseases-II

Azerbaijan Medical University,

Baku, Azerbaijan

Euroasian Journal of Hepato-Gastroenterology

